# DHA-eGCN: Differential Hyperedge Attention-Enhanced Graph Convolution Network for Skeleton-Based Human Action Recognition

**DOI:** 10.3390/s26123932

**Published:** 2026-06-20

**Authors:** Oskar Ika Adi Nugroho, Wen-Nung Lie

**Affiliations:** Department of Electrical Engineering and Advanced Institute of Manufacturing with High-Tech Innovations (AIM-HI), National Chung Cheng University, Chia-Yi 621, Taiwan; oskar@stikomyos.ac.id

**Keywords:** human action recognition, skeleton sequence, hyperedge, differential attention, graph convolution network, ensemble learning, NTU RGB+D, northwestern-UCLA

## Abstract

Skeleton-based human action recognition (HAR) requires models that preserve the local kinematic structure of the human body while capturing long-range spatiotemporal dependencies under noisy or incomplete joint observations. Traditional Graph Convolutional Networks (GCNs) provide topology-aligned inductive bias but are often limited by local information aggregation from neighboring joints. In contrast, attention-based mechanisms capture global interactions, yet they may attend to spurious correlations when skeletal constraints are weakly enforced. This paper proposes Differential Hyperedge Attention-enhanced GCN (DHA-eGCN), a hybrid architecture that couples structure-aware Differential Hyperedge Attention with multi-scale temporal convolution for spatiotemporal skeleton sequence processing. DHA injects skeletal structure into attention via hop-distance relative positional encoding and hyperedge context tokens generated via joint-to-part pooling. It further employs differential attention to suppress shared noisy correlations and enhance interaction selectivity. To strengthen spatial grounding, an explicit GCN branch is added under partial- or full-depth configurations, where the first four or all ten layers are applied with graph convolutions. The model further employs an ensemble strategy that combines predictions from multiple complementary model instances. Our experiments on NTU RGB+D 60 under the X-Sub and X-View protocols, NTU RGB+D 120 under the X-Sub and X-Set protocols, and Northwestern-UCLA demonstrate that DHA-eGCN consistently outperforms or remains competitive with strong graph-based, transformer-based, and hybrid state-of-the-art methods based on the same four-stream architecture. The best configuration achieves 93.7% and 97.0% on NTU RGB+D 60 X-Sub and X-View, respectively; 90.9% and 91.9% on NTU RGB+D 120 X-Sub and X-Set, respectively; and 97.6% on Northwestern-UCLA.

## 1. Introduction

Skeleton-based action recognition plays an important role in sensor-driven applications such as intelligent surveillance, healthcare monitoring, human–robot interaction, and smart environments. In these scenarios, skeleton data are typically obtained from RGB-D sensors or skeleton/pose-estimation systems, where robustness to noise, occlusion, and missing joints is critical for reliable sensing and decision-making. Recent sensor-oriented studies further show that visual sensors can estimate human keypoints in multi-person scenarios, providing structured pose representations that can support downstream human-centered analysis [1]. Therefore, improving the robustness and structural consistency of skeleton-based models is essential for real-world sensor applications.

**Figure 1:**
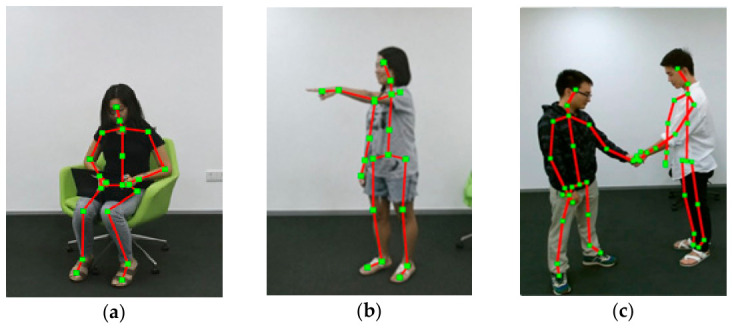
Illustrative examples of skeleton-based human action representation in the NTU RGB+D dataset. The figure shows two single-person actions and one two-person interaction. In human body graphs, the joints are represented as green nodes and bones are represented as red edges. The action types are (**a**) typing, (**b**) pointing to something, and (**c**) shaking hands.

Skeleton-based action recognition aims to classify human actions from 3D joint trajectories, typically obtained from RGB-D sensing pipelines (e.g., Kinect-like sensors) or pose-estimation systems [2]. In sensor-derived skeleton streams, measurement noises such as joint jitter, self-occlusion, and missing observations are common and can introduce spurious long-range correlations across time and joints. Driven by the growing demand for reliable human action understanding in real-world settings, intelligent video analytics has rapidly advanced toward more robust recognition in complex environments. Recent surveys highlight the growing importance of skeleton-based human activity recognition from RGB-D sensing [3]. In comparison with RGB-based cues, skeleton representations are compact and less sensitive to background clutter and appearance variations, making them attractive for efficient and privacy-friendly human motion understanding [4,5]. Despite these advantages, robust recognition remains challenging due to viewpoint changes, real-time execution requirements, and imperfect skeleton estimation from jitter, occlusion, and missing joints [4,5,6]. These difficulties demand models that can simultaneously encode local kinematic structure aligned with the human body and capture long-range spatiotemporal dependencies that reflect human action semantics. Figure 1 illustrates three examples of skeleton-based human action representation obtained from the NTU RGB+D dataset, showing how RGB-D sensor data can be converted into joint-node and bone-edge representations for action recognition.

**Figure 2:**
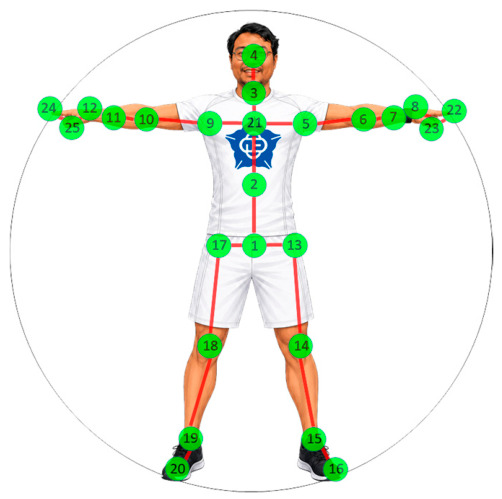
Skeleton graph representation of the human body. Each joint is modeled as a node (with index labels), and bones define the graph edges. Such structured representations are commonly obtained from RGB-D sensors (e.g., NTU RGB+D).

As shown in Figure 2, the human skeleton can be naturally modeled as a graph, where joints correspond to nodes and the connected bones define the edges. This graph structure provides the basis for topology-aware GCN and attention-based modeling. Human skeleton sequences naturally form spatiotemporal graphs, where temporal links connect joints between consecutive frames. Spatial–Temporal Graph Convolutional Networks (ST-GCN) [7] established a widely adopted formulation by modeling skeleton sequences as spatiotemporal graphs with both intra-frame skeletal links and inter-frame temporal connections, enabling hierarchical feature aggregation without handcrafted traversal rules. Building on this foundation, a large body of work [8,9,10,11] has proposed stronger GCN backbones through improved aggregation and topology modeling. Efficiency-focused formulations have also been explored to reduce computational overhead while maintaining competitive accuracy through shift-based operations [12]. Multi-scale adaptive designs further strengthen representation capacity by aggregating information at multiple temporal or spatial scales and adapting relation modeling across layers [13,14]. Overall, recent GCN designs increasingly combine adaptive topology, multi-scale aggregation, and global context modeling to improve recognition robustness [4,5,15].

While GCNs provide a strong inductive bias for local kinematic reasoning, many studies show that local aggregation alone can be insufficient for capturing global dependencies among distant joints, especially for actions involving cross-limb coordination. SelfGCN [16] explicitly combines graph convolution with self-attention to jointly model local and global relations. Transformer-style self-attention enables global dependency modeling but requires explicit structural priors and robustness mechanisms for noisy skeleton sequences. Differential Transformer proposes differential attention computed as the difference between two attention maps to suppress attention noise and encourage more selective interactions, supported by a structured parameterization and initialization strategy [17]. Topology-aware attention is also essential for skeleton action recognition. Recent advances further explore hypergraph modeling [18,19], reinforcing the motivation for higher-order and sample-adaptive relational reasoning. These developments align with survey findings that future progress will likely come from models that unify structure-aware relational inductive biases with strong global modeling and robust learning under imperfect skeleton observations [4,5].

Building on these observations, we design Differential Hyperedge Attention-enhanced GCN (abbreviated as DHA-eGCN) to unify topology-aware attention, differential noise suppression, and adaptive graph grounding. DHA-eGCN is implemented in two variants: DHA-eMGCN (or MaskedGCN) and DHA-eMAGCN (or Masked and Adaptive GCN). Both variants integrate three components. First, we employ a hyperedge-guided self-attention pathway to capture higher-order joint-group interactions, aligned with hypergraph-based skeleton transformers [18,19]. Second, we use multi-scale temporal convolutions (MSTCN) to capture short- and long-range motion patterns efficiently [7,12,13,20]. Third, we introduce a graph convolution branch with learnable edge-importance masking (i.e., MGCN), while in DHA-eMAGCN, we incorporate additional sample-dependent adaptive adjacency to capture action-conditioned relations [8,9,13,14,21]. Beyond backbone design, representation quality and fusion strategy are crucial for the final accuracy. Inspired by enhanced skeletal feature construction, we also design and evaluate a five-stream architecture with enriched higher-order joint features and demonstrate that selecting the best model combination across streams yields further gains through ensemble fusion [5,22].

This paper makes four contributions:

- We propose DHA-eGCN, a framework for skeleton-based action recognition that combines topology-guided hyperedge attention with differential attention to suppress shared noisy correlations under missing/jittery joint observations.
- We introduce two spatial graph modeling variants, MGCN and MAGCN, combining masked topology priors with optional sample-dependent adaptive adjacency for action-conditioned relation refinement, and evaluate both partial-GCN and full-GCN placement strategies.
- We study a five-stream implementation of DHA-eGCN with enriched skeletal feature inputs and confirm that our per-stream model selection strategy improves the performance of multi-stream ensemble learning with late fusion.
- On the NTU RGB+D 60, NTU RGB+D 120, and Northwestern-UCLA datasets, DHA-eGCN achieves competitive or superior Top-1 accuracy compared with recent state-of-the-art (SOTA) methods, reaching 93.7%/97.0% on NTU RGB+D 60 X-Sub/X-View, 90.9%/91.9% on NTU RGB+D 120 X-Sub/X-Set, and 97.6% on Northwestern-UCLA.

## 2. Related Work

### 2.1. GCN-Based Architectures

GCN-based methods model skeleton sequences as graphs and learn spatiotemporal representations by propagating information across joints and frames. ST-GCN [7], a pioneering development in this field, established the standard formulation by introducing spatiotemporal graph convolution over spatial kinematic links and temporal connections, becoming a common baseline for subsequent designs. A key limitation of early ST-GCN-style models is their fixed adjacencies between nodes/joints of the graph/skeleton, which may not fully capture action-specific relations among distant joints.

To address this, many works focus on adaptive topology learning. 2s-AGCN [8] introduces adaptive graph learning for adjacencies between nodes to complement fixed physical edges, enabling the graph structure to vary with the inputs and improving action-conditioned modeling. Multi-scale adaptation approaches extend this idea by jointly modeling relations at multiple node-hopping distances and adjusting aggregation patterns across network depth [13]. Topology refinement with structured gating has also been explored; for example, $G^{3}CN$ [14] refines topology using Gaussian refinement and gated graph convolution, reinforcing the value of learnable and structured adjacencies. Recent work has explored semantic-assisted topology refinement [23] to enhance structural awareness in GCN-based models. Another direction targets global dependency modeling beyond local neighborhoods. NLGCN [10] extends the receptive fields and integrates non-local graph reasoning to capture long-range joint interactions that are difficult to model with purely local aggregation alone. On the other hand, complementary designs might strengthen discriminability and training stability. For example, Richly Activated GCN (RA-GCN) [9] improves activation patterns and robustly highlights informative joints and relations; residual and dense-connection enhancements stabilize deep spatiotemporal graph learning and improve feature reuse [11]. Stronger GCN baseline studies further indicate that careful architectural engineering can yield models that are accurate, efficient, and more explainable, reducing the need for overly complex add-ons in some cases [20]. To address efficiency issues, Shift-GCN [12] reduces computational cost by replacing heavy graph operations with shift-based mechanisms while maintaining competitive accuracy. Recent lightweight temporal modeling approaches, such as TFC-GCN [24], emphasize cross-temporal feature extraction with gated filtering mechanisms to enhance temporal discrimination while reducing model complexity.

Robustness to imperfections in the skeleton is increasingly emphasized [6,21,25,26,27]. Action Jitter Killer [6] addresses joint noise through an explicit optimization cascade, showing that denoising and stabilizing skeleton observations can substantially improve recognition performance. DeGCN [21] further relaxes rigid neighborhood assumptions to complement global modeling by adopting deformable graph operations that adapt to complex pose variations and viewpoint changes.

Overall, surveys and systematic reviews summarize the evolution of GCN-based skeleton action recognition as moving toward adaptive, multi-scale, global-aware, and noise-robust graph modeling, while emphasizing optimization and evaluation protocols [4,5,15].

### 2.2. Transformer-Based Architectures

Transformer-based models introduce self-attention to capture long-range dependencies across joints and time. While global attention is expressive, skeleton sequences require topology awareness, and attention can be sensitive to noisy or irrelevant correlations, particularly when joints are missing or jittery. Hyperformer [19] injects topology priors into attention through hop-based relative positional encoding and incorporates hyperedge-aware interactions to model higher-order joint-group relations. These designs indicate that transformer-based skeleton recognizers benefit from explicitly encoding skeletal connectivity and body-part relations, rather than relying on generic token-to-token attention alone.

Attention robustness is increasingly important. Differential Transformer [17] proposes differential attention computed as the difference between two attention maps to suppress noise and encourage more selective interactions. This is relevant for skeleton-based action recognition where spurious correlations can arise from measurement noise, ambiguous poses, and partial observability [5,6]. Recent developments also extend transformers toward hypergraph adaptivity. Autoregressive Adaptive Hypergraph Transformer [18] explores adaptive hypergraph construction and refinement in an autoregressive framework, strengthening the case for higher-order and sample-adaptive relational reasoning. In addition, external knowledge and foundation-model guidance have been explored for low-data regimes. CrossGLG [28] leverages large language model (LLM) guidance for one-shot skeleton-based 3D action recognition, suggesting a growing interest in knowledge-assisted generalization. Reviews summarize that effective transformer-based models typically integrate structural priors and robustness strategies rather than relying on generic attention alone [4,5,15].

### 2.3. Hybrid GCN–Transformer Architectures

Hybrid models bridge graph and transformer paradigms by combining local graph inductive bias with global self-attention. SelfGCN [16] integrates graph convolution and self-attention to jointly capture local kinematic constraints and long-range dependencies, addressing the limits of purely local aggregation. Hyperformer [19] integrates hyperedge reasoning inside transformers via graph distance embedding to retain skeletal structure during training. Recent sensor-oriented work has also explored GCN-Transformer designs for human motion modeling, showing that graph convolution can capture spatial dependencies while Transformer modules strengthen temporal and interaction modeling [29].

This hybrid viewpoint is echoed in surveys that categorize many competitive skeleton recognizers as combinations of structured graph reasoning and global attention-like modeling [4,5].

### 2.4. Multi-Stream Ensemble Representations and Fusion

Multi-stream ensemble learning is widely used because different skeletal representations capture complementary cues. The classical four-stream setting (joint, bone, joint motion, and bone motion) remains a standard baseline and is frequently enhanced with late fusion for consistent gains [4,5,15]. Surveys emphasize that representation design and fusion strategy can be as important as backbone selection, particularly on large-scale datasets [4,5,15].

More recent works explore richer representations. Enhanced skeletal joint feature construction improves recognition by providing complementary spatial and temporal information beyond raw joints and bones [22]. Some methods try to improve discriminability by re-parameterizing or enriching input keypoints [28]. Noise-robust pipelines such as Action Jitter Killer [6] further motivate multi-stream modeling because complementary streams can mitigate stream-specific noise patterns. For multi-entity settings, CHASE [30] proposes adaptive shift strategies tailored to multi-person interactions, highlighting that representation and aggregation must remain stable when multiple skeleton instances interact in the same scene.

Efficient multi-modality self-supervision and masked modeling frameworks can also benefit multi-stream fusion where multiple streams differ in signal-to-noise ratio and semantic content [31,32]. Recent 3D point-cloud sequence studies further support the importance of representation fusion and motion decoupling for action recognition. SequentialPointNet^++^ [33] introduces an R-Hyperpoint representation that fuses global static pose units with regional dynamic motion-chain cues and then applies multi-phase temporal sub-action parsing to model heterogeneous temporal motion patterns. Similarly, MD-PCSN [34] proposes meta-motion decoupling for point-cloud sequences, where logarithmic spatiotemporal point convolution constructs multi-granularity motion units and a displacement-trajectory encoding module captures both short-term motion differences and long-range motion dependencies. Although these methods operate on point-cloud sequences rather than skeleton graphs, they reinforce the broader trend that effective human action recognition benefits from explicitly separating spatial structure, local motion evolution, and long-range temporal dependencies.

Overall, the literature supports evaluating richer stream decompositions together with stream-specific backbones and optimized fusion weights, since the most suitable inductive bias can vary by modality and noise characteristics [4,5,30,31,35].

### 2.5. Gap and Motivation

Despite steady progress, several gaps remain in skeleton-based action recognition. First, many GCN-based models rely on local aggregation and may still struggle to suppress spurious correlations caused by noisy or jittery joints, even when adaptive or non-local mechanisms are introduced [6,10]. While topology refinement and flexible operators improve modeling capacity, they do not directly address attention-level noise suppression and selective global interaction modeling, which is particularly relevant under ambiguous or partial observations [14,17,21]. Second, transformer-based models offer global dependency modeling and higher-order reasoning, but their effectiveness often depends on carefully injecting skeletal structure and controlling attention noise [17,18,19]. Third, hybrid designs show promise, yet there remains a need for architectures that balance stable structural priors with lightweight, sample-adaptive relational refinement, especially in early layers where spatial grounding is critical [8,16,21,32]. Finally, multi-stream fusion is widely beneficial, but different streams (with different input features) may favor different inductive biases; therefore, a uniform backbone choice for all streams may be suboptimal compared with stream-specific model selection and optimized fusion.

Motivated by these observations, we propose DHA-eGCN to integrate (i) multi-scale temporal convolution for efficient temporal modeling, (ii) hyperedge-guided attention to capture higher-order joint group relations, and (iii) a masked graph branch with optional adaptive adjacency to preserve topology priors while enabling action-conditioned refinement. In addition, by studying an enriched five-stream decomposition and cross-model selective ensemble, DHA-eGCN aims to explicitly leverage modality-specific complementarity to achieve stronger late-fusion performance.

## 3. Proposed Method

### 3.1. Problem Statement

Given *N* samples of skeleton sequences (e.g., in a mini-batch), where each is of *T* frames, *M* persons (e.g., for the NTU RGB+D dataset illustrated in Figure 1, we have *M* = 2), *V* joints (e.g., we have *V* = 25, as illustrated in Figure 2), and *C*-feature channels, we denote the raw tensor as

(1) $$X\in R^{N\times C\times T\times V\times M}.$$

**Figure 3:**
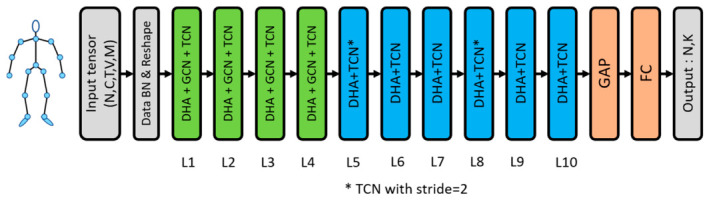
Overall architecture of the proposed DHA-eGCN, which has hybrid spatial (DHA + GCN) and temporal (TCN) modules. Here, we illustrate the partial-GCN setting, where the GCN branch is used only in layers 1–4, while the full-GCN setting applies the GCN branch to all ten layers. TCN is implemented in a multi-scale, where temporal down-sampling is performed using a stride of 2 at layers 5 and 8.

DHA-eGCN adopts a hierarchical spatiotemporal pipeline consisting of (i) data normalization and reshaping, (ii) a stack of *L* = 10 spatiotemporal blocks, and (iii) global averaging pooling (GAP) followed by a linear classifier, as illustrated in Figure 3. Each block couples a spatial module and a temporal module. The spatial module is built upon Differential Hyperedge Attention (DHA), which injects skeletal structure via hop-based relative positional encoding and hyperedge (part-level) tokens and suppresses spurious correlations via differential attention. The temporal module is implemented by a Multi-scale Temporal Convolution Network (MSTCN) to aggregate motion patterns over time.

DHA-eGCN supports two graph-branch placement configurations. As illustrated in Figure 3 for the partial-GCN setting, the explicit spatial GCN branch is applied only in layers 1–4, while layers 5–10 use DHA-only spatial modeling. In the full-GCN setting, the GCN branch is applied to all ten layers, allowing graph-based spatial reasoning to remain active throughout the backbone. These two configurations are evaluated in the ablation study, where the full-GCN setting achieves the best performance at the expense of a larger model size. Temporal down-sampling with stride 2 is applied at layers 5 and 8, following common practice in spatiotemporal backbones. Each GCN branch has two instantiations: MGCN (Masked GCN), which uses a learnable edge-importance mask on a fixed partitioned adjacency, and MAGCN (Masked and Adaptive GCN), which further introduces a sample-adaptive adjacency to refine relations conditioned on the input sequence.

### 3.2. Input Normalization and Data Layout

We follow prior skeleton-recognition pipelines and apply batch normalization over a concatenated person–joint–channel configuration to stabilize training [7]. Given the raw input ${X\in R}^{N\times C\times T\times V\times M}$, we permute and reshape it into ${X_{bn}\in R}^{N\times \left(M\cdot V\cdot C\right)\times T}$ so that all joints from all persons are concatenated along the channel dimension. We then apply normalization to the M·V·C channels to improve training stability and reshape the tensor back to a per-person layout. During backbone processing, the person dimension is merged into the batch dimension, yielding $\tilde{X}\in R^{B\times C\times T\times V}$, where B=N·M. We process each person’s instance independently within the backbone and fuse multi-person features only at the head via pooled feature averaging. Multi-person information is fused only at the output head by performing global pooling per person and then averaging the pooled features over the person dimension before classification.

### 3.3. DHA-eGCN Block

**Figure 4:**
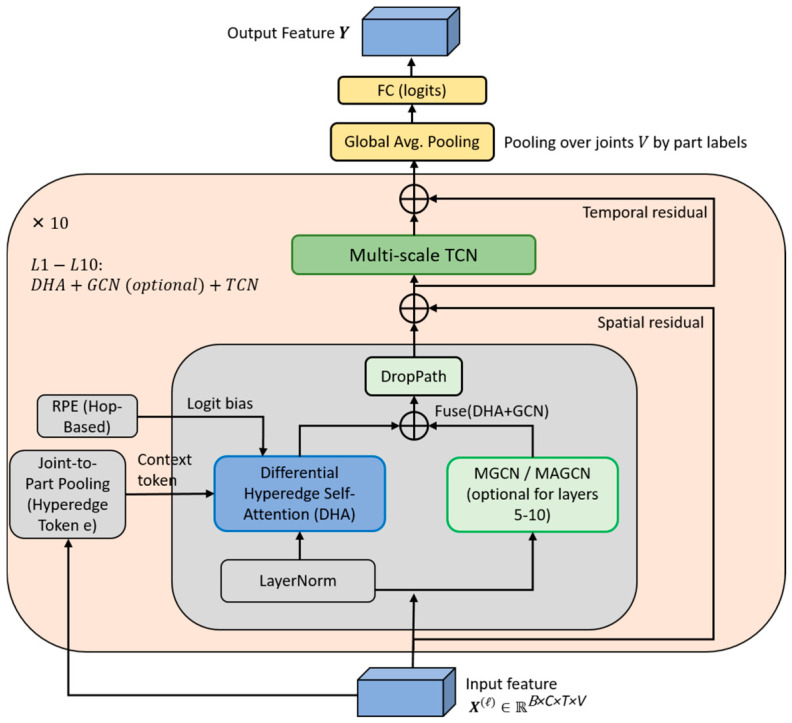
DHA-eGCN block. Each stage applies DHA for spatial modeling and multi-scale temporal convolution for temporal aggregation. An additional GCN branch is optionally fused with DHA according to the selected configuration (partial- or full-GCN). Hop-based RPE serves as a logit bias, while joint-to-part pooling provides hyperedge token context.

Each backbone stage is a spatiotemporal block that couples a spatial module and a multi-scale temporal module (Figure 4). Let $X^{\left(l\right)}\in R^{B\times C_{in}\times T\times V}$ denote the input feature map of the l-th block, where $C_{in}$ is the number of input feature channels. The spatial sub-layer first refines joint interactions using DHA, and it is augmented with an additional GCN branch according to the selected placement configuration, either partial-GCN or full-GCN. The temporal sub-layer then applies a multi-scale TCN to aggregate motion patterns over time using multiple dilated temporal convolution branches, producing the output $X^{\left(l+1\right)}$. This design combines topology-aware global spatial interaction with efficient temporal modeling, while maintaining the same tensor layout across all layers. We define the l-th block as

(2) $$Y^{\left(l\right)}={Res}_{l}^{sp}\;\left(X^{\left(l\right)}\right)+DropPath\left({Spatial\;}_{l}(X^{\left(l\right)})\;\right),$$

$$X^{\left(l+1\right)}=\;{TCN}_{l}\left(Y^{\left(l\right)}\right)+{Res}_{l}^{tcn}\left(Y^{\left(l\right)}\right),$$

where ${Spatial\;}_{l}\left(\cdot \right)=DHA(\cdot )$ or ${Spatial\;}_{l}\left(\cdot \right)=DHA\left(\cdot \right)+GCN(\cdot )$, depending on partial or full-GCN setting. The spatial residual mapping ${Res}_{l}^{sp}\left(\cdot \right)$ is an identity mapping when the input and spatial output have the same channel dimension; otherwise, a 1 × 1 convolutional projection is used for channel alignment. The temporal residual mapping ${Res}_{l}^{tcn}\left(\cdot \right)$ is used to match both the output channel dimension and temporal resolution, with stride $s_{l}\;\neq \;1$ applied at layers 5 and 8 for temporal down-sampling.

### 3.4. Spatial Module with Differential Hyperedge Attention (DHA)

#### 3.4.1. Structure-Aware Attention with Hop-Based RPE

To align self-attention with skeletal topology, DHA-eGCN adopts hop-based relative positional encoding (RPE) [19] derived from shortest-path distances on the physical skeleton graph, following the topology-injected attention design in Hyperformer [19]. We learn an embedding table R indexed by the hop distance between two joints *i* and *j*, *dist* (*i*,*j*), and inject the hop-specific embedding $R_{dist(i,j)}$ into attention logits as a topology-aware bias. Hop-based RPE encourages topology-consistent spatial reasoning by favoring relations supported by the skeleton structure while still allowing long-range interactions when beneficial.

#### 3.4.2. Hyperedge Tokens via Joint-to-Part Pooling

Our DHA-eGCN further incorporates hyperedge tokens to capture higher-order joint-group structure beyond pairwise relations, inspired by Hyperformer’s hyperedge-augmented attention (HyperSA) [19]. Specifically, joint features are pooled into part-level representations according to a predefined joint-to-part assignment (e.g., parts of the torso, left arm, right arm, left leg, right leg). Each part token summarizes body-part context and serves as a compact group representation.

To couple joints with these group tokens, the part tokens are broadcast back to joint positions by assigning each joint the token of its corresponding part label, forming a hyperedge-aligned context token for every joint. This joint-to-part and part-to-joint mechanism injects group-level context into attention computations through joint-to-hyperedge interactions. We emphasize that the group assignment is fixed and used to construct context tokens, rather than performing explicit hypergraph structure learning. As illustrated in Figure 4, the hyperedge token functions as a context signal within the attention module rather than a separate feature stream.

#### 3.4.3. Differential Hyperedge Attention Mechanism for Noise Suppression

Building on the Hyperformer-style structure-aware attention in each branch, DHA adopts a differential attention mechanism inspired by the Differential Transformer [17] to suppress noise-induced correlations. In skeleton sequences, measurement noise and imperfect observations (e.g., jitter and missing joints) can cause standard self-attention to allocate noticeable weight to irrelevant joints, which weakens spatial reasoning and reduces robustness [5,6].

**Figure 5:**
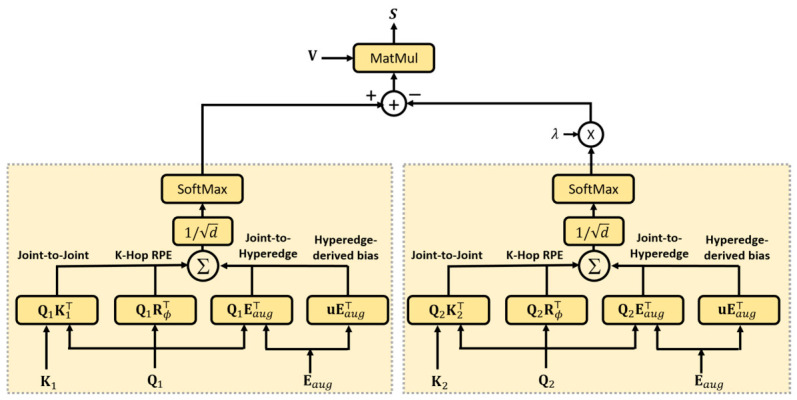
Differential Hyperedge Attention (DHA) architecture. Two parallel, structure-aware attention branches compute logits using four additive components. The final attention is formed by subtracting the second normalized map from the first using a learnable coefficient *λ* and then applied to the value features ***V***.

**Figure 6:**
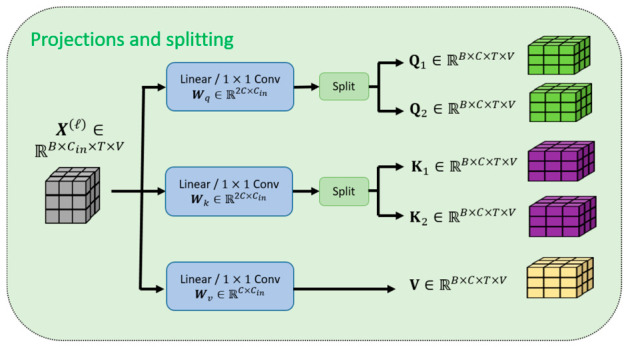
**V**, **Q**_1_, **K**_1_, **Q**_2_, **K**_2_ are calculated from the input features $X^{\left(l\right)}\in R^{B\times C_{in}\times T\times V}$ via linear projections ($W_{q},\;W_{k},and\;W_{v}$) and splitting.

Instead of producing a single attention map, DHA computes two parallel structure-aware attention maps (Figure 5) from the same input features $X^{\left(l\right)}$ using independent linear projections $W_{q},\;W_{k},and\;W_{v}$ and splitting (see Figure 6). Figure 5 illustrates the two-branch design. Both branches incorporate hop-based RPE and hyperedge context, so each attention map remains guided by skeletal topology and part-level structure. The final attention is formed by subtracting the second normalized map from the first with a learnable coefficient λ, which is modeled as a layer-specific subtraction coefficient within DHA to control the strength of the differential term without introducing excessive degrees of freedom.

As illustrated in Figure 5, let $q_{\left(b,i\right)}$ and $k_{\left(b,j\right)}$ denote the query and key vectors after linear projection from the input $X^{\left(l\right)}$ for branch $b\in \left\{1,2\right\}$ and joint pair *i* and *j*; $E_{aug,j}$ be the hyperedge-augmented token aligned to joint *j* (Section 3.4.2); $R_{\phi (i,j)}$ be the hop-based relative positional encoding indexed by hop distance ϕ(i,j) on the skeleton graph (Section 3.4.1); and the per-head dimension be *d*. We further introduce a learnable vector u to produce an additional scalar bias from $E_{aug,j}$. For branch *b*, we define the attention logit between joints *i* and *j* as [17]

$${IS}_{ij}^{\left(b\right)}={q_{\left(b,i\right)}k}_{\left(b,j\right)}^{⊤}+q_{\left(b,i\right)}E_{aug,j}^{⊤}+q_{\left(b,i\right)}R_{\phi \left(i,j\right)}^{⊤}+uE_{aug,j}^{⊤}$$

The branch-wise normalized attention map is then

$${\bar{S}}_{ij}^{\left(b\right)}={Softmax}_{j}\left(\frac{{IS}_{ij}^{\left(b\right)}}{\sqrt{d}}\right),\;b\in \left\{1,2\right\}\;$$

where ${Softmax}_{j}\left(\cdot \right)$ denotes normalization over all other joints *j* for a fixed query *i*. Finally, DHA forms the differential attention map as

(3) $$S_{ij}={V(\bar{S}}_{ij}^{\left(1\right)}-\lambda {\bar{S}}_{ij}^{\left(2\right)})$$

In Equation (3) and Figure 5, the attention logit for joint i attending to joint j is the sum of four components: (a) joint-to-joint relation, which captures pairwise joint dependencies based on feature similarity; (b) joint-to-hyperedge relation, which injects hyperedge (part-level) context into attention, enabling higher-order coupling or interactions beyond direct joint-to-joint matching; (c) hop-based RPE as a topology bias to introduce topology awareness by adding a hop-conditioned bias on the skeleton graph; and (d) a hyperedge-derived additive bias to provide an additional key-wise logit bias, which helps to stabilize and calibrate the influence of hyperedge information in the attention logits.

Based on this differential mechanism, correlations that persist across both maps are down-weighted, while discriminative interactions are preserved. In contrast, selective interactions that differ between two branches are retained and emphasized. As a result, DHA improves attention selectivity and reduces spurious long-range interactions that are weakly supported by the skeleton topology or part structure.

### 3.5. Graph Branch Placement

DHA-eGCN complements global spatial modeling with an explicit graph branch (Figure 4), motivated by the effectiveness of graph inductive bias and adaptive topology refinement in skeleton-based action recognition [8,9,10,13,21]. We consider two variants:

- MGCN: a masked GCN branch that learns edge-importance weights on top of a fixed partitioned adjacency (masked topology prior).
- MAGCN: a stronger masked plus adaptive GCN branch that additionally learns a sample-dependent adjacency to capture action-conditioned relations beyond physical bones [8,13].

To balance modeling capacity and efficiency, we consider two graph branch placement strategies. In the partial-GCN configuration, the graph branch is applied only in layers 1–4 to emphasize early spatial grounding. In the full-GCN configuration, the graph branch is applied to all ten layers, allowing graph-based spatial reasoning to persist throughout the backbone. While early layers benefit from strong topology priors for stabilizing low-level joint interactions, deeper layers also benefit from continued graph-based aggregation. As shown in the ablation study, extending the GCN branch to deeper layers consistently improves performance, indicating that graph-based spatial modeling remains complementary to DHA even at higher semantic levels.

#### 3.5.1. Masked GCN Branch (MGCN)

In DHA-eMGCN (i.e., DHA with MGCN), the GCN branch performs local spatial aggregation using a set of masked skeleton adjacencies while learning a soft edge-importance mask to reweight connections. Let $A\in R^{K\times V\times V}$ be the predefined adjacencies with K=3 spatial partitions for k-hop (*k* = 0,…, *K*−1) neighborhoods [7,8,16], and $M\in R^{K\times V\times V}$ be learnable edge-importance masks. Specifically, *k* = 0 represents the self-node, k = 1 specifies the neighboring nodes for each physical bone, and *k* = 2 collects neighbors accessible via a 2-hop distance. $A^{\left(k\right)}$ is a predefined and fixed joint-to-joint adjacency matrix based on the physical connectivity of the human skeleton, in which the element 1 indicates a connected joint pair, and 0 indicates a non-connected joint pair. The effective adjacency for the *k*-th partition is thus

(4) $${\bar{A}}_{M}^{\left(k\right)}=A^{\left(k\right)}⨀\;M^{\left(k\right)}$$

Given input features $X^{\left(l\right)}\in R^{B\times C\times T\times V}$, the branch aggregates neighbor information using ${\bar{A}}_{M}^{\left(k\right)}$, applies a partition-specific 1 × 1 projection, and then sums over all partitions:

(5) $$Z_{GCN}^{\left(l\right)}=\sigma \left(BN\left(\sum_{k=0}^{K\;-\;1}{Conv}^{\left(k\right)}\left(X^{\left(l\right)}{\bar{A}}_{M}^{\left(k\right)}\right)\right)\right),\;$$

where BN is the batch normalization and $\sigma \left(\cdot \right)$ denotes a nonlinear mapping. This masked topology prior preserves stable kinematic structure while allowing the model to emphasize task-relevant edges during training.

#### 3.5.2. Masked and Adaptive GCN Branch (MAGCN)

DHA-eMAGCN (i.e., DHA with MAGCN) strengthens the graph branch by augmenting the masked prior with a sample-dependent adjacency that captures action-conditioned relations beyond physical bones, consistent with adaptive graph modeling trends [8,13,14,21]. Specifically, we estimate an adaptive adjacency $E\left(X^{\left(l\right)}\right)\in R^{B\times V\times V}$ from the input features $X^{\left(l\right)}$, using lightweight projections. Let $\theta \left(\cdot \right)$ and ϕ(·) be 1 × 1 convolutions applied to a temporally pooled feature (e.g., time-mean), producing $\theta \left(X^{\left(l\right)}\right)\in R^{B\times V\times d}$ and $\phi \left(X^{\left(l\right)}\right)\in R^{B\times d\times V}$. The adaptive adjacency is obtained by

(6) $$E\left(X^{\left(l\right)}\right)=Softmax\left(\frac{\theta \left(X^{\left(l\right)}\right)\phi \left(X^{\left(l\right)}\right)}{\sqrt{d}}\right)$$

We blend this adaptive term with the masked prior using a learnable scalar γ initialized to zero:


(7) $${\bar{A}}_{MA}^{\left(k\right)}\left(X^{\left(l\right)}\right)={\bar{A}}_{M}^{\left(k\right)}+\gamma E\left(X^{\left(l\right)}\right).$$

The resulting message ${\bar{A}}_{MA}^{\left(k\right)}$ will be passed to Equation (5) for replacing ${\bar{A}}_{M}^{\left(k\right)}$ with ${\bar{A}}_{MA}^{\left(k\right)}$. This design retains the stability of the prior physical skeleton while enabling adaptive refinement and allowing the corresponding layers to better capture non-local, action-dependent joint relations.

### 3.6. Fusion of Global Attention and Local Graph Features

In layers where the GCN branch is enabled, DHA-eGCN combines global, topology-aware interactions from DHA with local, neighborhood aggregation from the GCN branch. Let $Z_{DHA}^{\left(l\right)}$ and $Z_{GCN}^{\left(l\right)}$ denote the outputs of DHA and the graph branch at layer *l*. We fuse them by simple summation and apply *DropPath* (.), which is based on stochastic depth (where residual layers or residual branches are randomly dropped during training and bypassed with the identity function) [36], before adding the residual connection:


(8) $$Y^{\left(l\right)}\;=\;X^{\left(l\right)}\;+\;DropPath\left(Z_{DHA}^{\left(l\right)}+Z_{GCN}^{\left(l\right)}\right).$$

Here, the residual for $X^{\left(l\right)}$ uses an identity mapping when dimensions match; otherwise, it is implemented by a 1 × 1 projection to align channels.

When the GCN branch is disabled, as in layers 5–10 of the partial-GCN setting, the spatial module becomes DHA-only:


(9) $$Y^{\left(l\right)}\;=\;X^{\left(l\right)}+DropPath\left(Z_{DHA}^{\left(l\right)}\right).$$

### 3.7. Multi-Scale Temporal Convolution Module

In DHA-eGCN, temporal dynamics are modeled by a Multi-scale Temporal Convolution (MSTCN) module that aggregates motion cues using multiple (denoted as *R*) parallel branches with different dilation rates, together with pooling-based branches (Figure 4). Given an input $Y^{\left(l\right)}\in R^{B\times C\times T\times V}$ from the output of the DHA spatial module (i.e., Equation (8) or (9)), each branch first applies a 1 × 1 projection to reduce channel dimension, followed by a temporal convolution with kernel size k and dilation d, producing features with different temporal receptive fields. Outputs from all the branches are concatenated along the channel dimension and combined with a residual connection:

(10) $${TCN}_{l}\left(Y^{\left(l\right)}\right)=Concat\left({\left\{B_{l,r}\left(Y^{\left(l\right)}\right)\right\}}_{r=1}^{R}\right),$$

where $B_{l,r}$ denotes the r-th temporal branch (dilated conv or pooling).

DHA-eGCN performs stride-2 temporal down-sampling at layers 5 and 8 to increase the effective temporal receptive field while reducing computational cost. This hierarchical design preserves fine-grained short-term motion patterns in early layers and progressively captures longer-range dynamics in deeper layers, which is particularly important for complex actions with extended temporal structure.

### 3.8. Output Head and Person Aggregation

After the final backbone layer, DHA-eGCN applies global average pooling over the temporal and joint dimensions to obtain a clip-level representation for each person instance. Let the final feature map be $X^{\left(L\right)}\in R^{B\times C\times T\times V}$. The per-person pooled feature is

(11) $$f={GAP}_{t,v}\left(X^{\left(L\right)}\right)\in R^{B\times C},$$

where ${GAP}_{t,v}\left(\cdot \right)$ denotes average pooling over time *t* and joints *v*.

To aggregate multiple persons, we reshape f into $R^{N\times M\times C}$ (since B=N·M) and average over the person dimension:


(12) $$\bar{f}\;=\;\frac{1}{M}\sum_{m=1}^{M}f_{:,m,:}\in R^{N\times C}.$$

Finally, a linear classifier maps the aggregated feature to *K* action classes:

(13) $$\hat{y}\;=\;\bar{f}W^{⊤}+b,\hat{y}\in R^{N\times K}$$

where $W\in R^{K\times C}$ and $b\in R^{N\times K}$ are learnable parameters.

### 3.9. Multi-Stream and Late Fusion

Beyond the classical four-stream setup [37,38] (joint, bone, joint motion, bone motion) widely used in ST-GCN [7] pipelines and summarized in recent reviews [4,5], we evaluate an enriched five-stream decomposition {J,E,S,M,A} (RICH5), inspired by enhanced skeletal joint feature construction from our prior work [22]. Let $p_{t,j}\in R^{3}$ denote the 3D coordinate of joint *j* at time *t*, and let *r* be the root (spine) joint. We first apply subtraction to each joint $p_{t,j}$ to become a root-centered (normalized) or relative joint ${\tilde{p}}_{t,j}\;=\;p_{t,j}\;-\;p_{t,r}$.

#### 3.9.1. Vector-Based Joint Features (J/E/S/M/A) for Ensemble Learning

**Figure 7:**
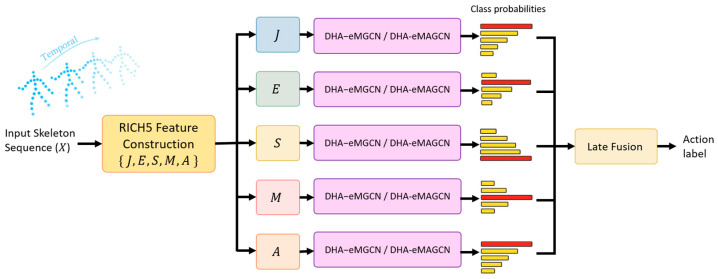
Multiple-stream DHA-eGCN architecture based on RICH5 (J, E, S, M, A) decomposition.

**Figure 8:**
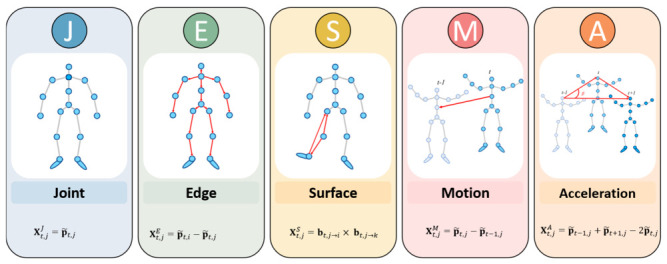
Enriched five-stream (RICH5) decomposition for skeleton-based action recognition. Illustration of the proposed vector-based decomposition into five vectors: Joint (J), Edge (E), Surface (S), Motion (M), and Acceleration (A).

Following the enriched skeletal feature construction in [22], we build five complementary streams. Each stream is fed with different joint features from {*J*, *E*, *S*, *M*, *A*} as the input, which is then individually processed by two variants, DHA-eMGCN or DHA-eMAGCN, as illustrated in Figure 7. The three spatial features {J,E,S} correspond to $\left\{S_{1},S_{2},S_{3}\right\}$ in [19], while the two temporal features {M,A} correspond to $\left\{T_{2},T_{3}\right\}$ in [22]. The {*J*, *E*, *S*, *M*, *A*} features are visualized in Figure 8 and explained below.

(1) Joint stream *J* (1st-order spatial feature).

We define a directed 3D vector from the root to joint *j* by:


(14) $$X_{t,j}^{J}\;=\;{\tilde{p}}_{t,j}$$

(2) Edge stream *E* (2nd-order spatial feature).

Let N(j) be the neighbors of joint *j* in the skeleton graph. A directed bone vector from joint *j* to the neighboring joint *i* is defined as

(15) $$X_{t,j}^{E}={\tilde{p}}_{t,i}-{\tilde{p}}_{t,j},\;\;\;i\in N(j)$$

This ordered joint pair (*j* ⟶ *i*) is selected such that the bone vector $X_{t,j\to i}^{E}\;$ points radially outwards away from the root joint.

(3) Surface stream S (3rd-order spatial feature).

For a joint triplet $\left(i,j,k\right),$ where i,k∈N(j), we define

(16a) $$b_{t,j\to i}={\tilde{p}}_{t,i}-{\tilde{p}}_{t,j},\;\;b_{t,j\to k}={\tilde{p}}_{t,k}-{\tilde{p}}_{t,j}\;$$

and calculate an oriented vector that represents the normal of the plane defined by (*i*, *j*, *k*)-joints using the cross product:


(16b) $$X_{t,j}^{S}=b_{t,j\to i}\times \;b_{t,j\to k}.$$

This information encodes the angle formed by i-j-k joints, capturing a higher-order geometric relation that complements joints and bones. For joints with only one neighbor and the root (spine) joint, which has more than two neighbors, the two neighboring joints *i* and *k* are specifically assigned.

(4) Motion stream M (2nd-order temporal feature).

We define a joint-motion vector as

(17) $$X_{t,j}^{M}={\tilde{p}}_{t,j}-{\tilde{p}}_{t-1,j}$$

This complements the spatial information by emphasizing motion dynamics, which is important for actions with similar poses but different temporal patterns [22].

(5) Acceleration stream *A* (3rd-order temporal feature).

Using three consecutive instants (t − 1,t,t + 1), we define

(18) $$X_{t,j}^{A}=\left({\tilde{p}}_{t-1,j}-{\tilde{p}}_{t,j}\right)-\left({\tilde{p}}_{t,j}-{\tilde{p}}_{t+1,j}\right)={\tilde{p}}_{t-1,j}+{\tilde{p}}_{t+1,j}-2{\tilde{p}}_{t,j},$$

which reflects trajectory curvature and provides acceleration-like cues useful for actions involving speed or direction changes [22]. Boundary frames are handled by padding or replication so that all streams share the same temporal length.

In comparison with the traditional four-stream configuration (i.e., joint, bone, joint motion, and bone motion), the proposed RICH5 joint feature decomposition presents higher-order information derived from more joints (spatially or temporally). For example, “J”, “E”, “S”, “M”, and “A” correspond to spatial order 1, 2, and 3 and temporal order 2 and 3, respectively [22], while traditional joint, bone, joint motion, and bone motion correspond to spatial and temporal orders of at most 2. In view of this, RICH5 is more promising for modeling dynamic joint behaviors for action recognition.

#### 3.9.2. Late Fusion and Cross-Stream Model Selection

Each stream s∈{J,E,S,M,A} is processed by a dedicated DHA-eGCN model, using either DHA-eMGCN or DHA-eMAGCN architecture. We adopt late fusion via a weighted sum of class probability outputs from all streams:

(19) $$\hat{g}=\sum_{s\in \left\{J,E,S,M,A\right\}}\alpha_{s}g_{s},\;\;\;\alpha_{s}\geq 0,$$

where $g_{s}$ is the predicted class probability vector from stream *s*, and $\alpha_{s}$ is the fusion weight for the corresponding stream *s*. Late fusion follows the common score-level fusion practice in multi-stream skeleton action recognition [4,5,15]. In our experiments, we evaluate the offline grid-searched fusion weights to analyze the complementarity among different streams and backbone variants.

For the RICH5 setting, we further conduct an offline multi-model ensemble analysis by selecting among stream-specific DHA-eMGCN and DHA-eMAGCN candidates. This analysis evaluates up to $2^{5}=32$ candidate model combinations to study whether different streams benefit from different graph-branch variants. This procedure searches over multiple candidate models and fusion configurations based on the test set of NTU RGB+D 60 and might risk overfitting to the test distribution. For stricter practical deployment, the ensemble configuration should be selected using a separate validation subset split from the training data and then fixed before final test-time evaluation. In the following experiments, we provide additional evidence that the multi-model ensemble configuration selected on NTU RGB+D 60 can be generalized to other benchmarks, including NTU RGB+D 120 and Northwestern-UCLA.

## 4. Experiments

### 4.1. Experimental Settings

We evaluate the proposed method on three widely used skeleton-based action recognition benchmarks: NTU RGB+D 60 [39], NTU RGB+D 120 [40], and Northwestern-UCLA [41]. NTU RGB+D 60 contains 56,880 skeleton sequences from 60 action classes, captured by three Kinect v2 cameras, and is commonly evaluated under the Cross-Subject (X-Sub) and Cross-View (X-View) protocols. NTU RGB+D 120 is an extended and more challenging version of NTU RGB+D 60, containing 113,945 sequences from 120 action classes, with a larger number of subjects and more diverse acquisition setups. It is commonly evaluated under the Cross-Subject (X-Sub) and Cross-Setup (X-Set) protocols. Therefore, NTU RGB+D 120 provides a broader and more difficult benchmark for evaluating the generalization ability of skeleton-based action recognition models. Northwestern-UCLA [41] is a cross-view action recognition dataset containing RGB, depth, and human skeleton data captured by three Kinect cameras. It includes 10 action categories performed by 10 actors and is commonly evaluated under the Cross-View setting, where samples from two camera views are used for training and samples from the remaining camera view are used for testing. We report Top-1 classification accuracy under the standard evaluation protocols of each dataset, namely X-Sub and X-View for NTU RGB+D 60, X-Sub and X-Set for NTU RGB+D 120, and Cross-View for Northwestern-UCLA. Reporting Top-1 accuracy under standard benchmark protocols follows the common practice in skeleton-based action recognition studies [4,5,15]. For each stream in the multi-stream ensemble architecture, either DHA-eMGCN or DHA-eMAGCN is used based on the model-selection ensemble configuration (see the results in Table 1 and Table 2).

All models were implemented using Python 3.9.13 and PyTorch 2.7.1 [42], and trained for 140 epochs using cross-entropy loss. The learning rate is initialized to 0.025 and decayed by a factor of 0.1 at epochs 110 and 120 [43]. For the experiments on NTU RGB+D 60 and NTU RGB+D 120, we adopt the same training setting: a batch size of 32 and an input sequence length of 64 frames. Data preprocessing follows the pipeline provided by [44]. Unless otherwise stated, all experiments use a 10-layer backbone with 324 feature channels. Training is conducted on an NVIDIA GeForce RTX 4090 GPU (CUDA 12.6, driver 560.28.03).

**Table 1:** Top-1 accuracy on NTU RGB+D 60, NTU RGB+D 120, and Northwestern-UCLA under standard evaluation protocols.

Architecture	Method	Number of Streams	NTU RGB+D 60		NTU RGB+D 120		NW-UCLA
			X-Sub (%)	X-View (%)	X-Sub (%)	X-Set (%)	
Graph Convolution	ST-GCN [7]	1	81.5	88.3	-	-	-
	2S-AGCN [8]	2	88.5	95.1	-	-	-
	SelfGCN [16]	4	93.1	96.6	89.4	91.0	96.8
	Shift-GCN [12]	4	90.7	96.5	85.9	87.6	94.6
	SGN [44]	1	89.0	94.5	72.9	81.5	-
	TFC-GCN [24]	1	87.9	91.5	83.0	81.6	-
	CTR-GCN [23]	4	92.4	96.8	88.9	90.6	96.5
	Info-GCN [43]	4	93.0	97.1	89.8	91.2	97.0
	HLP-GCN [45]	4	92.7	96.9	89.0	90.8	96.8
	HD-GCN [46]	6	93.4	97.2	90.1	91.6	97.2
	BlockGCN [47]	4	93.1	97.0	90.3	91.5	96.9
Transformer	ST-TR [48]	2	87.1	91.8	-	-	-
	DSTA [49]	4	91.5	96.4	86.6	89.0	-
	Hyperformer [19]	4	92.9	96.5	89.9	91.3	96.7
	SkateFormer [50]	4	93.5	**97.8**	89.8	91.4	**98.3**
Hybrid Model (GCN + Att)	Dynamic GCN [51]	4	91.5	96.0	87.3	88.6	-
	EfficientGCN-B4 [52]	3	92.1	96.1	88.7	88.9	-
	FCSA-GCN [53]	4	93.6	97.5	90.5	91.3	97.2
	DHA-eGCN (ours, RICH4, full-GCN)	4	**93.7**	97.0	**90.9**	**91.9**	97.6

**Table 2:** Progressive performance improvements on NTU RGB+D 60.

Backbone Architecture	Partial/Full-GCN	Multi-Stream Ensembles	GCN Model	Top-1 X-Sub (%)	Top-1 X-View (%)
Hyperformer [19]	N/A	STD4	N/A	92.8	96.5
DHA-eMGCN	Partial	STD4	MGCN	93.1	96.8
DHA-eMAGCN	Partial	STD4	MAGCN	93.2	96.7
DHA-eMGCN	Full	STD4	MGCN	93.4	96.8
DHA-eMAGCN	Full	STD4	MAGCN	93.4	96.8
DHA-eMGCN	Partial	RICH4	MGCN	93.0	96.7
DHA-eMAGCN	Partial	RICH4	MAGCN	93.3	96.6
DHA-eMGCN	Full	RICH4	MGCN	93.4	96.8
DHA-eMAGCN	Full	RICH4	MAGCN	93.5	96.9
DHA-eMGCN	Partial	RICH5	MGCN	93.1	96.7
DHA-eMAGCN	Partial	RICH5	MAGCN	93.4	96.9
DHA-eMGCN	Full	RICH5	MGCN	93.4	96.8
DHA-eMAGCN	Full	RICH5	MAGCN	93.6	96.9
DHA-eGCN with multi-model ensemble selection (*)	Partial	RICH4	Selection (J, E, S, M): (MA, M, M, MA)	93.4	96.7
DHA-eGCN with multi-model ensemble selection (*)	Partial	RICH5	Selection (J, E, S, M, A): (MA, M, MA, MA, MA)	93.4	96.7
DHA-eGCN with multi-model ensemble selection (*)	Full	RICH4	Selection (J, E, S, M): (MA, M, M, MA)	93.7	97.0
DHA-eGCN with multi-model ensemble selection (*)	Full	RICH5	Selection (J, E, S, M, A): (MA, M, MA, MA, MA)	93.7	97.0

Re-implemented by us (the original paper reported 92.9%). * Two-model selection per stream. For RICH4, there are 2^4^ = 16 combinations; for RICH5, there are 2^5^ = 32 combinations.

We evaluate the standard four-stream setting (joint, bone, joint motion, bone motion), the five-stream RICH5 decomposition [22], and a variant four-stream RICH4. Multi-stream recognition is performed via late fusion, implemented as a weighted sum of per-stream class probability vectors.

### 4.2. Comparison with State-of-the-Art (SOTA) Approaches

We compare DHA-eGCN with representative GCN-based, transformer-based, and hybrid skeleton-based action recognition methods reported on NTU RGB+D 60 [39], NTU RGB+D 120 [40], and Northwestern-UCLA [41]. DHA-eGCN is trained separately on the training split of each dataset. For the multi-model ensemble setting, the stream-specific model configuration is determined through the offline ensemble analysis on NTU RGB+D 60, as discussed in Section 4.3 and Table 2, and the selected configuration is then applied to NTU RGB+D 120 and Northwestern-UCLA without additional re-selection. Table 1 summarizes the Top-1 accuracy under the standard evaluation protocols of the three datasets. The results of the compared SOTA methods are reported as in their original papers.

On NTU RGB+D 60, the best DHA-eGCN configuration achieves 93.7% on X-Sub and 97.0% on X-View. The X-Sub result is higher than those of recent GCN-based, transformer-based, and hybrid methods listed in Table 1, while the X-View result remains competitive with strong SOTA approaches. On the larger NTU RGB+D 120 dataset, DHA-eGCN achieves 90.9% on X-Sub and 91.9% on X-Set, outperforming the compared methods in both protocols. These results indicate that DHA-eGCN is effective not only on NTU RGB+D 60 but also on the more challenging NTU RGB+D 120 benchmark, which contains more action categories, subjects, and acquisition setups. A more detailed analysis is provided in Section 4.3 and Section 4.7. On Northwestern-UCLA, DHA-eGCN achieves 97.6% Top-1 accuracy using the RICH4 full-GCN setting. This result is highly competitive with recent state-of-the-art methods and outperforms most compared approaches, including Hyperformer [19] and SelfGCN [16]. It is lower only than SkateFormer [50], which reports 98.3%. Since Northwestern-UCLA is evaluated under a cross-view protocol and contains fewer training samples than NTU RGB+D, this result provides additional evidence that the proposed Differential Hyperedge Attention and graph grounding remain effective under viewpoint variation and limited-data conditions.

For Hyperformer [19], we report our re-implemented X-Sub accuracy of 92.8% on NTU RGB+D 60, whereas the original paper reported 92.9%. This minor 0.1% discrepancy is within normal re-implementation variation and may be attributed to differences in the random seed, software environment, preprocessing, or training details. We explicitly indicate this distinction in the table footnote for transparency.

### 4.3. Ablation Study

We perform controlled ablations to isolate the contributions of (i) the DHA and MSTCN backbone, (ii) enriched RICH-style joint feature decomposition, (iii) graph-branch placement, and (iv) model selection among multiple streams for late fusion.

Following prior work, we use Hyperformer [19] with the standard four-stream (STD4) setting as the baseline for comparison in Table 2 and report the Top-1 accuracy. Replacing the backbone with DHA-eGCN while keeping the same four-stream inputs yields consistent gains: DHA-eMGCN achieves 93.1%, and DHA-eMAGCN further improves to 93.2% compared to the Hyperformer backbone [19] (92.8%), indicating that DHA with masked graph refinement and optional sample-adaptive adjacency improves recognition without changing the input representation. Replacing the partial-GCN placement with the full-GCN further advances the accuracy to 93.4% for both eMGCN and eMAGCN.

Next, we replace the standard 4-stream formulation with enriched RICH-style streams. For afair comparison, we provide two settings: RICH4 containing J/E/S/M streams and RICH5 [22] containing all J/E/S/M/A streams. Under RICH4, the single-model setting achieves 93.0% and 93.3% for partial-GCN DHA-eMGCN and DHA-eMAGCN, respectively. With full-GCN, the performance improves to 93.4% and 93.5%. Extending to RICH5 further improves the best single-model result to 93.6% with full-GCN DHA-eMAGCN. This suggests that the enriched geometric and dynamic cues provide complementary information and that the acceleration-like stream (i.e., A) can add useful temporal dynamics to the motion stream (M), especially for actions with similar poses but different movement patterns.

To investigate the impact of graph convolution placement, we compare the partial-GCN design (layers 1–4) with the full-GCN variant (layers 1–10) across the STD4, RICH4, and RICH5 settings. As shown in Table 2, applying the GCN branch across all layers consistently improves or maintains performance over restricting it to early layers. For example, under RICH4, DHA-eMAGCN improves from 93.3% in the partial-GCN setting to 93.5% in the full-GCN setting. Under RICH5, DHA-eMAGCN improves from 93.4% to 93.6%. These results indicate that extending graph convolution to deeper layers enhances spatial modeling capacity, allowing the network to capture richer joint interactions even at higher semantic levels. This suggests that combining DHA with persistent graph reasoning provides complementary benefits throughout the network.

From Table 2, it is also observed that enabling stream-wise model selection between DHA-eMGCN and DHA-eMAGCN further improves late fusion. In this setting, each stream can select either the MGCN or MAGCN variant before weighted score-level fusion. For RICH4, there are 2^4^ = 16 possible combinations, while for RICH5, there are 2^5^ = 32 possible combinations. Under the full-GCN setting, DHA-eGCN with multi-model ensemble selection reaches 93.7% on X-Sub and 97.0% on X-View for both RICH4 and RICH5. For example, in RICH4, the selected configuration is (MA, M, M, MA) for the (J, E, S, M) streams, indicating that MAGCN is selected for the joint and motion streams, while MGCN is selected for the edge and surface streams. In the RICH5 setting, the selected configuration is (MA, M, MA, MA, MA) for the (J, E, S, M, A) streams, respectively. These results suggest that different input modalities may benefit from different spatial inductive biases, and that enforcing a single uniform backbone across all streams can be suboptimal.

The reported result of 93.7% is obtained from a post-training multi-model ensemble selection analysis based on the test set. The NTU RGB+D 60 benchmark provides predefined training and testing splits but does not provide an official validation split for selecting ensemble configurations. Therefore, this multi-model ensemble selection result should be interpreted as an analysis of stream and model complementarity rather than as a validation-selected deployment protocol. For a stricter practical deployment setting, the ensemble configuration should be selected using a held-out validation subset split from the training data and then fixed before final evaluation on the official test set.

To overcome this deficiency, we adopt the same multi-model ensemble selection configuration (i.e., (MA, M, M, MA) for the (J, E, S, M) streams in RICH4) and test the NTU RGB+D 120 with the indicated configuration after the four streams have been trained based on the NTU RGB+D 120 training set. From the results shown in Table 1, we achieve 90.9% and 91.9% for the X-Sub and X-Set protocols, which also outperform the SOTA methods. This then indicates that the multi-model ensemble selection based on the NTU RGB+D 60 dataset can be further generalized to the larger-scale test set of NTU RGB+D 120.

### 4.4. Computational Complexity Analysis

To evaluate the computational efficiency of DHA-eGCN, we report the number of trainable parameters, giga floating-point operations (GFLOPs), inference time, and Top-1 accuracy in Table 3. For multi-stream ensemble settings, the total computational cost is obtained by summing the costs of all stream-specific models because each stream must be evaluated before score-level late fusion. The overhead of late fusion itself is negligible because it only performs a weighted summation of class likelihood scores.

As shown in Table 3, the full-GCN variants require higher computational cost than the partial-GCN variants because the graph branch is applied to all layers. Among the single-stream models, DHA-eMAGCN full-GCN achieves the best accuracy, with 92.0% on X-Sub and 95.6% on X-View, using 11.96 M parameters, 20.86 GFLOPs, and 10.94 ms/sample. The RICH4 full-GCN ensemble further improves the accuracy to 93.7% on X-Sub and 97.0% on X-View. However, since the ensemble combines two DHA-eMGCN full-GCN streams and two DHA-eMAGCN full-GCN streams, its total cost increases to 46.88 M parameters, 83.40 GFLOPs, and 42.22 ms/sample. Therefore, the single-stream architecture is more suitable for lower-cost deployment, especially with the early fusion version proposed in [22], whereas the RICH4 ensemble is preferable when recognition accuracy is prioritized.

### 4.5. Analysis of the Learned Differential Coefficient λ

To examine whether the differential attention mechanism is used by the trained model, we extract the final learned differential coefficient λ (see Figure 5 or Equation (3)) from the best DHA-eMAGCN full-GCN design and report its layer-wise values. The learned λ values vary across layers 1–10: (−1.90, −2.91, 0.76, −6.61, 0.76, 0.76, 0.76, 0.76, 0.76, 0.76). Layers 1, 2, and 4 produce negative values, while the remaining layers retain positive values. Obviously, the learned λ values do not collapse to zero across layers, indicating that the second attention map contributes to the final differential attention computation. According to Equation (3), a positive λ strengthens the subtractive behavior between the two attention maps, whereas a negative λ effectively changes the second attention map into an additive correction term. This suggests that the model adaptively controls not only the magnitude but also the direction of the interaction between the two attention maps at different network depths. In particular, the larger absolute values in the early layers indicate that differential attention is more strongly adjusted during low-level spatial feature extraction, where skeleton noise and local joint ambiguity are more prominent.

### 4.6. Missing-Joint Robustness Analysis

To address the concern regarding robustness to missing or imperfect skeleton observations, we conducted an additional testing-time random missing-joint masking experiment, as reported in Table 4. In this experiment, both Hyperformer and DHA-eMAGCN full-GCN were evaluated using the joint stream on the NTU RGB+D 60 X-Sub protocol. The models were trained using clean skeleton sequences, and no retraining was performed for the missing-joint settings. During inference, 10%, 20%, and 30% of the joints were randomly selected from each skeleton sequence and masked by setting their 3D coordinates to zero. This setting simulates incomplete skeleton inputs caused by occlusion, pose-estimation failure, or sensor noise.

As shown in Table 4, the proposed DHA-eMAGCN full-GCN consistently outperforms Hyperformer under all missing-joint ratios. Under the clean setting, DHA-eMAGCN full-GCN achieves 92.0% accuracy, compared with 90.7% for Hyperformer. When 10%, 20%, and 30% of joints are randomly masked, DHA-eMAGCN full-GCN obtains 88.0%, 84.6%, and 77.4% accuracy, respectively, while Hyperformer drops to 85.7%, 81.5%, and 72.6%. The performance gap becomes larger as the missing-joint ratio increases, from 1.3% in the clean setting to 2.3%, 3.1%, and 4.8% under 10%, 20%, and 30% joint dropping, respectively. These results indicate that DHA-eMAGCN full-GCN is more robust to incomplete skeleton inputs and better preserves discriminative action representations under missing-joint perturbations.

### 4.7. Discussion

The experimental results show that DHA-eGCN achieves competitive performance across datasets with different scales and evaluation protocols. It obtains 93.7%/97.0% on NTU RGB+D 60 X-Sub/X-View and 90.9%/91.9% on NTU RGB+D 120 X-Sub/X-Set, indicating that the proposed framework remains effective on the larger and more challenging NTU RGB+D 120 benchmark. On the smaller cross-view Northwestern-UCLA dataset, DHA-eGCN achieves 97.6% using the same RICH4 full-GCN ensemble selection, outperforming most compared methods and remaining slightly below SkateFormer [50]. These results suggest that the proposed topology-aware Differential Hyperedge Attention and graph-enhanced stream fusion are not limited to the NTU RGB+D dataset family.

The ablation results indicate that the performance gain comes from the combination of the DHA-eGCN backbone and the enriched stream representation. The DHA module improves spatial modeling by combining hop-aware topology bias, hyperedge context, and differential attention, while the GCN branch provides additional graph-based grounding. Meanwhile, the RICH-style stream decomposition introduces complementary geometric and motion cues beyond standard joint, bone, and motion streams. The multi-model ensemble selection results further suggest that different streams may benefit from different graph variants, making stream-specific model selection more flexible than using one uniform backbone for all input modalities.

The computational analysis shows a clear trade-off between recognition accuracy and model cost. Full-GCN variants generally improve accuracy compared with partial-GCN variants, but they require more parameters and GFLOPs. Similarly, the RICH4 full-GCN ensemble achieves the best overall accuracy, but its computational cost increases because multiple stream-specific models must be evaluated before late fusion. Therefore, the multi-stream ensemble setting is more suitable when accuracy is prioritized, whereas single-stream or early-fusion settings, such as the early fusion version proposed in [22], are more appropriate for lower-cost deployment scenarios.

The missing-joint robustness analysis further supports the stability of DHA-eGCN under incomplete skeleton observations. DHA-eMAGCN full-GCN consistently outperforms Hyperformer [19] when 10%, 20%, and 30% of joints are randomly dropped during inference, and the performance gap becomes larger as the missing-joint ratio increases. This suggests that Differential Hyperedge Attention and graph grounding help preserve discriminative skeleton representations when part of the joint information is corrupted or missing.

## 5. Conclusions

We present a DHA-eGCN architecture that integrates (i) topology-informed, hyperedge-aware attention with hop-based relative positional encoding, (ii) differential attention to suppress noisy correlations and improve selectivity, and (iii) a masked graph branch with an optional sample-adaptive adjacency refinement. We investigated two practical variants, DHA-eMGCN (MaskedGCN) and DHA-eMAGCN (Masked+Adaptive GCN), and further examined both partial-GCN and full-GCN configurations. The ablation results show that extending the GCN branch to all ten layers provides the best overall performance, indicating that persistent graph-based spatial modeling remains complementary to DHA throughout the backbone.

Experiments on NTU RGB+D 60, NTU RGB+D 120, and Northwestern-UCLA demonstrate the effectiveness and generalization capability of DHA-eGCN. On NTU RGB+D 60, DHA-eGCN with RICH4, full-GCN, and multi-model ensemble selection achieves 93.7% on X-Sub and 97.0% on X-View. On the larger NTU RGB+D 120 dataset, the same configuration achieves 90.9% on X-Sub and 91.9% on X-Set. Furthermore, on Northwestern-UCLA, DHA-eGCN achieves 97.6% accuracy under the cross-view evaluation protocol, providing additional evidence that the proposed framework generalizes to another skeleton-based benchmark with different camera viewpoints and limited training samples. Additional analyses also show the computational trade-off of the multi-stream ensemble setting, the behavior of the learned differential coefficient, and improved robustness under testing-time missing-joint perturbations.

Future work will extend this evaluation to more realistic corruption settings, such as structured occlusion, joint jitter, and confidence-aware skeleton inputs. Another promising direction is to combine DHA-eGCN with incomplete-skeleton imputation methods, such as the GCN-based imputation and action recognition framework in [26], allowing missing joints to be reconstructed before recognition instead of being directly masked to zero. In addition, lightweight fusion strategies, such as uncertainty-aware weighting or gating, remain important for reducing computational cost while preserving recognition accuracy in real-time applications.

## Figures and Tables

**Table 3:** Computational complexity and Top-1 accuracy of DHA-eGCN variants on NTU RGB+D 60.

Method	Stream	Params (M)	GFLOPs	Inference Time (ms/Sample)	Top-1	
					X-Sub (%)	X-View (%)
DHA-eMGCN partial	Joint	9.58	17.81	9.06	91.5	95.4
DHA-eMAGCN partial	Joint	9.73	17.82	9.34	91.6	95.5
DHA-eMGCN full-GCN	Joint	11.48	20.84	10.17	91.9	95.6
DHA-eMAGCN full-GCN	Joint	11.96	20.86	10.94	92.0	95.6
DHA-eGCN (ours, RICH4, full-GCN)	4	46.88	83.40	42.22	93.7	97.0

Note: GFLOPs and inference time are measured with input tensor size 1 × 3 × 64 × 25 × 2 (N, C, T, V, M).

**Table 4:** Top-1 accuracy under testing-time random missing-joint masking on NTU RGB+D 60 X-Sub using only the joint stream.

Model	Clean	Drop 10%	Drop 20%	Drop 30%
Hyperformer [19]	90.7	85.7	81.5	72.6
DHA-eMAGCN full-GCN	92	88	84.6	77.4

Note: In the missing-joint settings, 10%, 20%, and 30% of joints were randomly masked during inference by setting their 3D coordinates to zero. No retraining was performed.

## Data Availability

The code and configuration files are available at github.com/oskar-ika-adi-nugroho/DHA-eGCN. The datasets used in this study are publicly available from their respective providers, including NTU RGB+D 60, NTU RGB+D 120, and Northwestern-UCLA, and are subject to the original licenses and access terms.

## References

[1] Fincato M., Vezzani R. (2025). DualPose: Dual-Block Transformer Decoder with Contrastive Denoising for Multi-Person Pose Estimation. Sensors.

[2] Lie W.N., Vann V. (2024). Estimating a 3D Human Skeleton from a Single RGB Image by Fusing Predicted Depths from Multiple Virtual Viewpoints. Sensors.

[3] Iadarola G., Mengarelli A., Iarlori S., Monteriù A., Spinsante S. (2025). RGB-D Cameras and Brain-Computer Interfaces for Human Activity Recognition: An Overview. Sensors.

[4] Liu M., Liu H., Hu Q., Ren B., Yuan J., Lin J., Wen J. (2025). 3D skeleton-based action recognition: A review. arXiv.

[5] Liu Y., Liu R., Hu Y., Wu M., Xin W., Miao Q., Wu S., Li L. (2025). A Systematic Review of Skeleton-Based Action Recognition: Methods, Challenges, and Future Directions. IEEE Trans. Neural Netw. Learn. Syst..

[6] Liu R., Liu Y., Xin W., Miao Q., Li L. (2024). Action jitter killer: Joint noise optimization cascade for skeleton-based action recognition. IEEE Trans. Instrum. Meas..

[7] Yan S., Xiong Y., Lin D. Spatial temporal graph convolutional networks for skeleton-based action recognition. Proceedings of the AAAI Conference on Artificial Intelligence.

[8] Shi L., Zhang Y., Cheng J., Lu H. Two-stream adaptive graph convolutional networks for skeleton-based action recognition. Proceedings of the IEEE/CVF Conference on Computer Vision and Pattern Recognition.

[9] Song Y.F., Zhang Z., Shan C., Wang L. (2020). Richly activated graph convolutional network for robust skeleton-based action recognition. IEEE Trans. Circuits Syst. Video Technol..

[10] Shi L., Zhang Y., Cheng J., Lu H. (2018). Non-local graph convolutional networks for skeleton-based action recognition. arXiv.

[11] Wu C., Wu X.J., Kittler J. Spatial residual layer and dense connection block enhanced spatial temporal graph convolutional network for skeleton-based action recognition. Proceedings of the IEEE/CVF International Conference on Computer Vision Workshops.

[12] Cheng K., Zhang Y., He X., Chen W., Cheng J., Lu H. Skeleton-based action recognition with shift graph convolutional network. Proceedings of the IEEE/CVF Conference on Computer Vision and Pattern Recognition.

[13] Hu H., Fang Y., Han M., Qi X. (2024). Multi-scale adaptive graph convolution network for skeleton-based action recognition. IEEE Access.

[14] Ren H., Luo Z., Fan H., Yuan X., Wang G., Zhang L. G^3^ CN: Gaussian Topology Refinement Gated Graph Convolutional Network for Skeleton-Based Action Recognition. Proceedings of the IEEE/RSJ International Conference on Intelligent Robots and Systems.

[15] Chung J.L., Ong L.Y., Leow M.C. (2025). A systematic literature review of optimization methods in skeleton-based human action recognition. IEEE Access.

[16] Wu Z., Sun P., Chen X., Tang K., Xu T., Zou L., Wang X., Tan M., Cheng F., Weise T. (2024). SelfGCN: Graph convolution network with self-attention for skeleton-based action recognition. IEEE Trans. Image Process..

[17] Ye T., Dong L., Xia Y., Sun Y., Zhu Y., Huang G., Wei F. (2024). Differential transformer. arXiv.

[18] Ray A., Raj A., Kolekar M.H. Autoregressive adaptive hypergraph transformer for skeleton-based activity recognition. Proceedings of the IEEE/CVF Winter Conference on Applications of Computer Vision.

[19] Zhou Y., Cheng Z.Q., Li C., Fang Y., Geng Y., Xie X., Keuper M. (2022). Hypergraph transformer for skeleton-based action recognition. arXiv.

[20] Song Y.F., Zhang Z., Shan C., Wang L. Stronger, faster and more explainable: A graph convolutional baseline for skeleton-based action recognition. Proceedings of the 28th ACM International Conference on Multimedia.

[21] Myung W., Su N., Xue J.H., Wang G. (2024). DeGCN: Deformable graph convolutional networks for skeleton-based action recognition. IEEE Trans. Image Process..

[22] Lie W.N., Nugroho O.I.A. Improving Graph-Convolution-Network-Based Action Recognition Through Enhanced Skeletal Joint Features. Proceedings of the International Conference on Consumer Electronics-Taiwan.

[23] Chen Y., Zhang Z., Yuan C., Li B., Deng Y., Hu W. Channel-wise topology refinement graph convolution for skeleton-based action recognition. Proceedings of the IEEE/CVF International Conference on Computer Vision.

[24] Wang K., Deng H. (2023). TFC-GCN: Lightweight temporal feature cross-extraction graph convolutional network for skeleton-based action recognition. Sensors.

[25] Hu H., Cao Y., Fang Y., Meng Z. (2025). Semantics-assisted training graph convolution network for skeleton-based action recognition. Sensors.

[26] Lie W.N., Lin Y.Y., Chiang J.C. Imputation and Action Recognition of Incomplete Human Skeletons Based on Graph Convolutional Neural Network. Proceedings of the 10th IEEE International Conference on Communications and Electronics (IEEE ICCE).

[27] Lie W.N., Le K.T., Vann V., Chiang J.C., Bui N.D. Skeleton-Sequence-Based Early Action Recognition by Using Graph Convolutional Neural Networks and Knowledge Distillation Techniques. Proceedings of the Asia Pacific Signal and Information Processing Association Annual Summit and Conference.

[28] Yan T., Zeng W., Xiao Y., Tong X., Tan B., Fang Z., Cao Z., Zhou J.T. Crossglg: Llm guides one-shot skeleton-based 3D action recognition in a cross-level manner. Proceedings of the European Conference on Computer Vision.

[29] Šajina R., Oreški G., Ivašić-Kos M. (2025). GCN-Transformer: Graph Convolutional Network and Transformer for Multi-Person Pose Forecasting Using Sensor-Based Motion Data. Sensors.

[30] Wen Y., Liu M., Wu S., Ding B. (2024). CHASE: Learning convex hull adaptive shift for skeleton-based multi-entity action recognition. Adv. Neural Inf. Process. Syst..

[31] Wei Y., Peng K., Roitberg A., Zhang J., Zheng J., Liu R., Chen Y., Yang K., Stiefelhagen R. Elevating skeleton-based action recognition with efficient multi-modality self-supervision. Proceedings of the IEEE International Conference on Acoustics, Speech and Signal Processing.

[32] Wu S., Lu G., Han Z., Chen L. (2025). A robust two-stage framework for human skeleton action recognition with GAIN and masked autoencoder. Neurocomputing.

[33] Li X., Chen Z., Gao G., Qi L., Ye Q., Zhao M. (2026). SequentialPointNet++: A Reinforced-Hyperpoint Network through Pose and Motion-chain Fusion for 3D Action Recognition. Inf. Fusion.

[34] Li X., Gao G., Chen Z., Li X., Huang Q. (2026). MD-PCSN: Meta-Motion Decoupling Point Cloud Sequence Network for Privacy-Preserving Human Action Recognition in AI Machines. IEEE Trans. Netw. Serv. Manag..

[35] Yang Y., Zhang J., Zhang J., Du B., Tu Z. (2025). Expressive Keypoints for Skeleton-Based Action Recognition via Progressive Skeleton Evolution. IEEE Trans. Image Process..

[36] Huang G., Sun Y., Liu Z., Sedra D., Weinberger K.Q. Deep networks with stochastic depth. Proceedings of the European Conference on Computer Vision.

[37] Zhou Y., Xu T., Wu C., Wu X., Kittler J. Adaptive hyper-graph convolution network for skeleton-based human action recognition with virtual connections. Proceedings of the IEEE/CVF International Conference on Computer Vision.

[38] Shi L., Zhang Y., Cheng J., Lu H. (2020). Skeleton-based action recognition with multi-stream adaptive graph convolutional networks. IEEE Trans. Image Process..

[39] Shahroudy A., Liu J., Ng T.T., Wang G. NTU RGB+D: A Large Scale Dataset for 3D Human Activity Analysis. Proceedings of the IEEE Conference on Computer Vision and Pattern Recognition.

[40] Liu J., Shahroudy A., Perez M., Wang G., Duan L.-Y., Kot A.C. (2020). NTU RGB+D 120: A Large-Scale Benchmark for 3D Human Activity Understanding. IEEE Trans. Pattern Anal. Mach. Intell..

[41] Wang J., Nie X., Xia Y., Wu Y., Zhu S.-C. Cross-view Action Modeling, Learning and Recognition. Proceedings of the IEEE Conference on Computer Vision and Pattern Recognition (CVPR).

[42] Paszke A., Gross S., Massa F., Lerer A., Bradbury J., Chanan G., Killeen T., Lin Z., Gimelshein N., Antiga L. Pytorch: An imperative style, high-performance deep learning library. Proceedings of the Advances in Neural Information Processing Systems.

[43] Chi H.G., Ha M.H., Chi S., Lee S.W., Huang Q., Ramani K. Infogcn: Representation learning for human skeleton-based action recognition. Proceedings of the IEEE/CVF Conference on Computer Vision and Pattern Recognition.

[44] Zhang P., Lan C., Zeng W., Xing J., Xue J., Zheng N. Semantics-guided neural networks for efficient skeleton-based human action recognition. Proceedings of the IEEE/CVF Conference on Computer Vision and Pattern Recognition.

[45] Wei C., Deng Z. Accommodating self-attentional heterophily topology into high-and low-pass graph convolutional network for skeleton-based action recognition. Proceedings of the International Joint Conference on Neural Networks.

[46] Lee J., Lee M., Lee D., Lee S. Hierarchically decomposed graph convolutional networks for skeleton-based action recognition. Proceedings of the IEEE/CVF International Conference on Computer Vision.

[47] Zhou Y., Yan X., Cheng Z.-Q., Yan Y., Dai Q., Hua X.-S. BlockGCN: Redefine Topology Awareness for Skeleton-Based Action Recognition. Proceedings of the IEEE/CVF Conference on Computer Vision and Pattern Recognition (CVPR).

[48] Plizzari C., Cannici M., Matteucci M. Spatial temporal transformer network for skeleton-based action recognition. Proceedings of the International Conference on Pattern Recognition.

[49] Shi L., Zhang Y., Cheng J., Lu H. Decoupled spatial-temporal attention network for skeleton-based action-gesture recognition. Proceedings of the Asian Conference on Computer Vision.

[50] Do J., Lee M., Lee D., Lee S. SkateFormer: Skeletal-Temporal Transformer for Human Action Recognition. Proceedings of the European Conference on Computer Vision (ECCV).

[51] Ye F., Pu S., Zhong Q., Li C., Xie D., Tang H. Dynamic GCN: Context-enriched topology learning for skeleton-based action recognition. Proceedings of the 28th ACM International Conference on Multimedia.

[52] Song Y.F., Zhang Z., Shan C., Wang L. (2023). Constructing stronger and faster baselines for skeleton-based action recognition. IEEE Trans. Pattern Anal. Mach. Intell..

[53] Kilic U., Oztimur Karadag O., Tumuklu Ozyer G. (2026). Fine-to-Coarse Self-Attention Graph Convolutional Network for Skeleton-Based Action Recognition. Appl. Soft Comput..

